# Ultra Short Echo Time MRI of Iron-Labelled Mesenchymal Stem Cells in an Ovine Osteochondral Defect Model

**DOI:** 10.1038/s41598-020-64423-4

**Published:** 2020-05-21

**Authors:** Joshua D. Kaggie, Hareklea Markides, Martin J. Graves, James MacKay, Gavin Houston, Alicia El Haj, Fiona Gilbert, Frances Henson

**Affiliations:** 1 Department of Radiology, Box 218, University of Cambridge, Cambridge, United Kingdom; 2 Cambridge University Hospitals NHS Foundation Trust, Addenbrooke’s Hospital, Cambridge, United Kingdom; 3 Institute of Science and Technology in Medicine, Guy Hilton Research Centre, Keele University, Thornburrow Drive, Stoke-on-Trent, ST4 7QB UK; 4 Department of Chemical Engineering, Healthcare Technologies Institute, Birmingham University, B15 2TT Birmingham, UK; 5 GE Healthcare, Amersham, United Kingdom; 6 Division of Trauma and Orthopaedic Surgery, University of Cambridge, Cambridge, United Kingdom; 7 Comparative Musculoskeletal Biology Group, Department of Veterinary Medicine, University of Cambridge, Cambridge, United Kingdom

**Keywords:** Mesenchymal stem cells, Bone, Stem-cell research

## Abstract

Multipotent Mesenchymal Stem/Stromal Cells (MSCs) are widely used in cellular therapy for joint repair. However, the use of MSC therapies is complicated by a lack of understanding of the behaviour of cells and repair within the joint. Current methods of MSC tracking include labelling the cells with Super Paramagnetic Iron Oxide nanoparticles (SPIOs). However, standard acquisition sequences (T_2_ and T_2_*) give poor anatomical definition in the presence of SPIOs. To avoid anatomical compromise in the presence of SPIOs, we have investigated the use of Ultra-short Echo Time (UTE) MRI, using a 3D cones acquisition trajectory. This method was used to track SPIO labelled MSC injected into joints containing osteochondral defects in experimental sheep. This study demonstrates that multiple echo times from UTE with 3 T MRI can provide excellent anatomical detail of osteochondral defects and demonstrate similar features to histology. This work also monitors the location of SPIO-labelled cells for regenerative medicine of the knee with MRI, histology, and Prussian blue staining. With these methods, we show that the SPIOs do not hone to the site of defect but instead aggregate in the location of injection, which suggests that any repair mechanism with this disease model must trigger a secondary process.

## Introduction

Osteoarthritis (OA) is the most common disease affecting the synovial joints. OA causes significant welfare and economic burden in both animal and human healthcare^1^. A significant cause of OA is articular joint surface defects (OA). New strategies for tissue engineering may be able to influence joint surface defect healing^2–10^. These strategies include cell based therapies and small molecular treatments, which have been widely used in regenerative medicine^2–10^. The development of these methods have, however, been hampered by the difficulty in probing their underlying mechanisms. New technology is required to investigate the mechanisms by which joint repair occurs, particularly imaging technology for the whole-joint investigation of cellular treatments.

Multipotent Mesenchymal Stromal/Stem Cells (MSCs) are progenitor cells, which can be procured from numerous tissues in the body, such as fat and bone marrow^11^. MSCs can develop into musculoskeletal cells, such as osteocytes, chondrocytes, and adipocytes^12,13^. Whilst MSCs have been used widely in the treatment of joint disease^14,15^, the mechanism of action for MSCs remain an area of research and debate. Proposed mechanisms of MSC repair include: paracrine action for recruiting nearby cells; exosome/microvesicle or tunneling nanotube exchange for differentiation into replacement cell types; and differentiation and integration within the resident tissues^16,17^.

Irrespective of the mechanism of action of MSC, there is a significant body of research that demonstrates the efficacy of MSC in treating such joint pathology as meniscal regeneration^18^ and osteoarthritis^19,20^. These results indicate that intra-articular MSC injections could represent a genuine therapeutic strategy for joint disease. However, the successful launch of MSC therapy as a therapeutic treatment is complicated by a lack of understanding of cellular behaviour within the joint. The lack of sensitive functional tools that can be used to measure cells after injection impedes *in vivo* cell fate tracking, especially within deep tissues.

Cellular tracking can be done *in vivo* by labelling the cells, injecting them into the joint, and monitoring them using specific imaging techniques^21^. The successful interpretation of these cell tracking experiments relies on the sensitivity and specificity of the imaging modality. Magnetic Resonance Imaging (MRI) of cells labelled with superparamagnetic iron-oxide (SPIO) particles has been shown to detect single cells^22,23^, and thereby enable tracking of MSC infiltration^24–27^. The tracking of SPIO-labelled MSCs has been performed in many tissues including the myocardium, liver, and the central nervous system^6–10^, as well as in joints^2–5^. SPIOs enable cellular tracking by increasing the R_2_ and R_2_* relaxivities in MRI, compared to normal tissue^10^. SPIO-labelled MSC tracking in musculoskeletal regions is complicated by both the fast (=short) signal relaxation times of both bone and SPIOs, which creates signal loss with standard MRI sequences. A high concentration of SPIOs can reduce the MRI signal decay, or transverse relaxation times, sufficiently to make tissue completely invisible^10,28^. The challenge is the visualisation of these tissues and their subsequent repair in these bony regions.

A method for visualising SPIO-induced signal loss and bone structure is to use an MRI readout with UTE k-space sampling, such as 3D cones^29^. 3D cones uses three dimensional spiral acquisition trajectories that are interleaved to efficiently image a volume^29^. 3D cones can also be acquired with different TEs to enable quantitative R_2_^*^ (=1/T_2_*) mapping. A 3D cones readout can improve the SPIO labelled MSCs, whereas standard Cartesian k-space readouts may result in poor image quality. The aim of this study was to evaluate the use of 3D cones MRI sequences for SPIO detection and for visualizing subsequent joint changes after the introduction of SPIO-labelled MSCs in an experimentally created osteochondral defect in a large animal preclinical model.

## Results

Both histology and Prussian Blue staining confirmed the presence of SPIOs in the fat pad and surrounding synovial fluid, where there was significant MRI signal decay. MSCs were not detected in other regions with any of these methods, specifically, at the site of the defect where MSCs were believed to hone. The investigation of this MSC and OA model suggests that MSCs do not differentiate, otherwise they would hone to the defect. If these MSCs are involved in repair, the mechanism of action would be in the recruitment of secondary cells.

We used three quantitative MRI techniques that showed no effect of the MSCs on R_2_* or R_2_ quantitation outside of the fat pad. Amongst these techniques, we used a UTE sequence with five echo times or repeats of the k-space trajectory within a single repetition time (TR) for quantification of R_2_*. The UTE sequence also enabled visualisation within bone in the presence of SPIOs.

### Surgery

The surgical procedures and animal recovery were uneventful. The post-mortem joints did not demonstrate osteophyte formation, nor joint degeneration outside of the defect.

### Localization of SPIOs in histological sections

**Figure 1:**
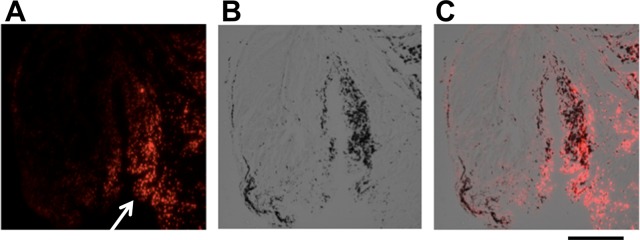
Synovial membrane from the joint of a sheep five weeks after the creation of an osteochondral defect: SPIO labelled MSC injection occurred four weeks after defect creation; animal sacrifice occurred one week following the injection of SPIO labelled MSCs. These sections show the presence of SPIOs (white arrow) in the synovial membrane with (**A**) fluorescence microscopy, (**B**) Prussian Blue staining (monochrome image), and (**C**) as a merged image. Scale bar = 75 μm.

Fluorescent microscopy and Prussian Blue staining were used to localize SPIOs within the joint. No cell labelled SPIOs could be detected in the osteochondral defects in any of the animals studied. However, SPIOs were detected within the synovium in all animals (Fig. 1).

### Localization of focal injection of SPIOs

**Figure 2:**
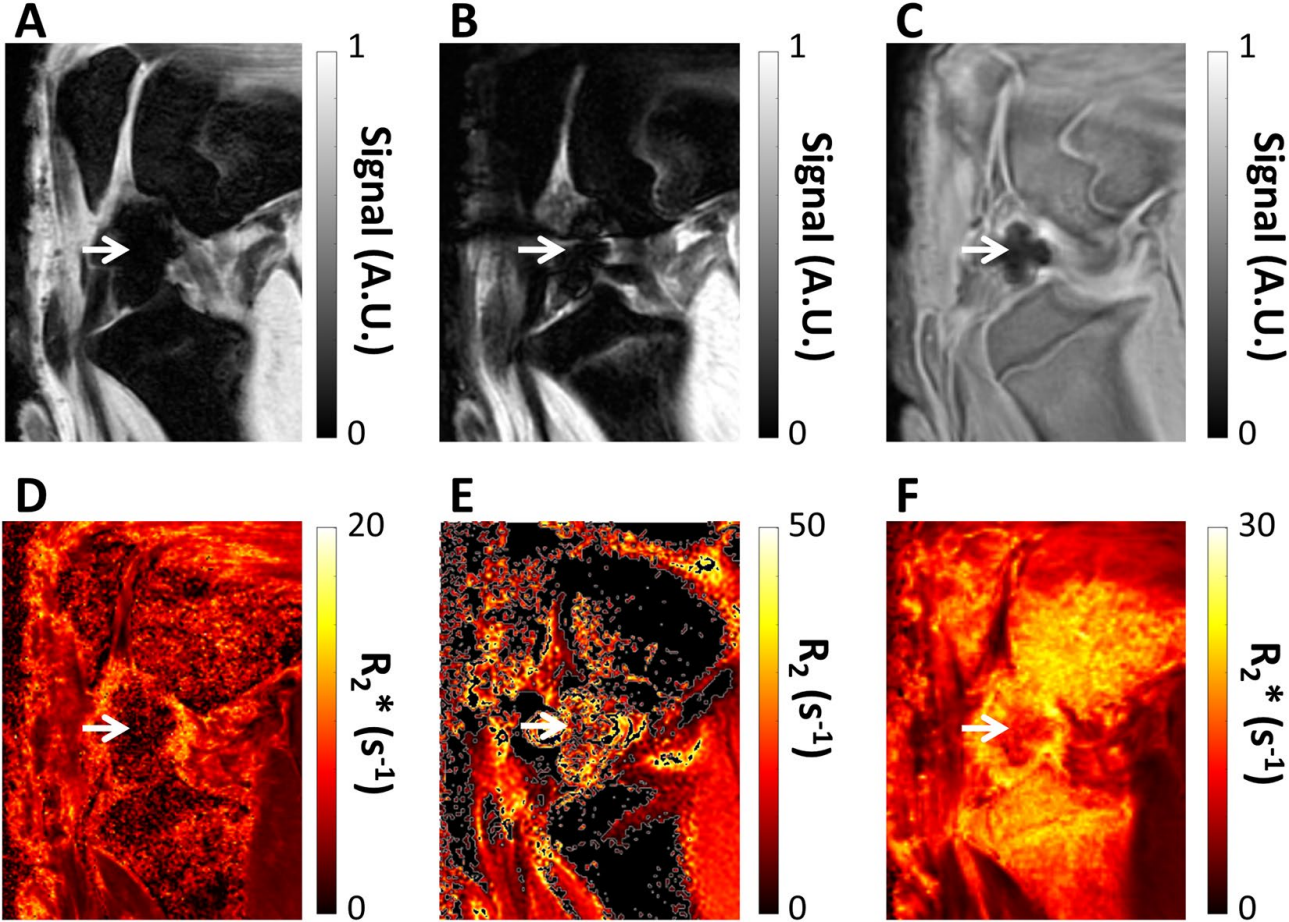
Magnetic resonance images (**A**–**F**) relaxation time maps. Images (**A**,**B**,**D**,**E**) were obtained with standard Cartesian sequences; images (**C**,**F**) with 3D cones. Images acquired from the same specimen showing the appearance of a focal injection of SPIO-labelled MSCs into the suprapatella fat pad of an ovine stifle. The SPIOs are seen as a blooming artifact in the cranial aspect of the femoral-tibial joint in both images (white arrows).

MRIs of the site were obtained using a standard 3D cones UTE and Cartesian mGE acquisitions, following a focal injection of SPIO-labelled MSC into the suprapatellar fat pad of the stifle joint, are shown in Fig. 2. These images demonstrate heavy signal attenuation and a blooming artefact, indicating the presence of high concentrations of SPIOs. The high SPIO concentration causes a much larger loss of anatomical definition in the Cartesian mGE acquisition, whilst in the 3D cones image significantly more anatomical detail is seen. Signal relaxivity rates were not calculated in these areas with significant blooming, as the signal at the first TE was not sufficient to be able to obtain an appropriate fit.

### Effect of SPIO-labelled MSC on R_2_*

**Figure 3:**
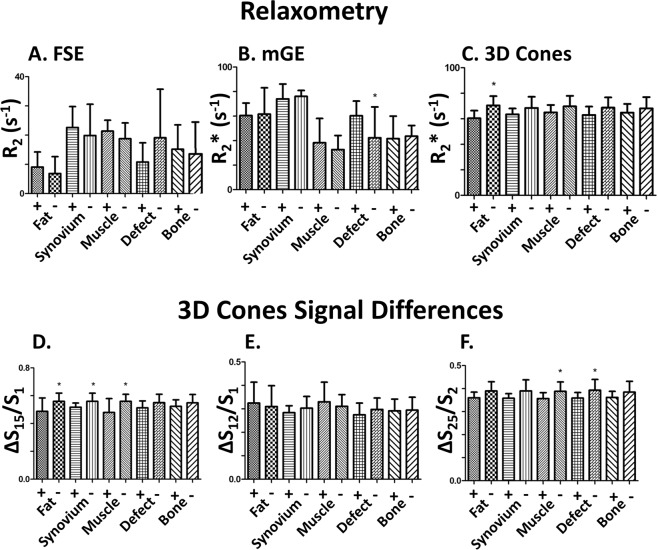
Summary of the mean relaxivity rates (where the top of each bar indicates the mean) and standard deviations (indicated in one direction by the ‘T’ beyond each bar plot). These are listed for (**A**) R_2_ with FSE, (**B**) R_2_* with a Cartesian mGE, (**C**) R_2_* with multi-echo 3D Cones, and signal loss ratios of 3D cones between (**D**) the first and second echo, (**E**) the first and fifth echo, and the (**F**) the second and fifth echo. A plus ‘+’ indicates the measurements in joints injected with SPIOs; a negative ‘−’ indicates the lack of SPIOs; a star ‘*’ indicates if the t-test p-value was below 0.15.

**Figure 4:**
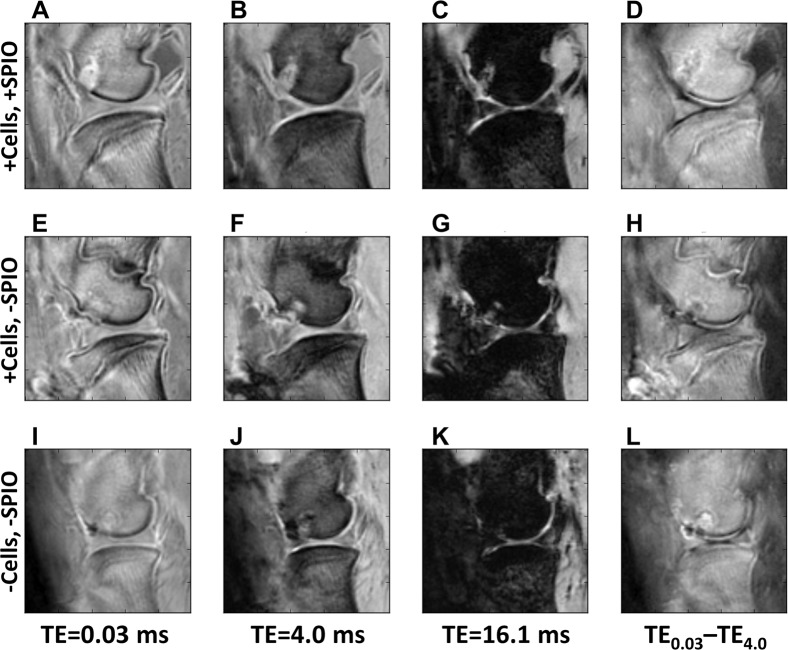
Ovine stifles imaged with 3D cones four weeks post creation of an osteochondral defect in the distal femoral condyle. (**A**–**D**) Joints that received an injection of SPIO-labelled MSCs 1 week post-surgery; (**E**–**H**) Joints that received an injection of unlabelled MSCs 1 week post-surgery; (**I**–**L**) Joints that did not have MSCs injected. (**A**,**E**,**I)** were imaged at TE = 0.03 ms; (**B**,**F**,**J)** were imaged at TE = 4.0 ms; (**C**,**G**,**K)** were imaged at TE = 16.1 ms. (**D**,**H**,**L)** represents the subtraction image of the first two TEs, highlighting the difference between (**A**,**E**,**I**,**B**,**F**,**J**). The defect has either similar or higher signal intensity than bone in the single echo time/column (**A**,**E**,**I**), regardless of method. The introduction of SPIOs do not cause increased defect decay, which would make the image in (**C**) darker.

Quantification of signal was performed in order to determine the effect of the presence of SPIO labelled MSC in the stifle joints in 3D cones acquired images. In the stifles in which SPIO labelled MSCs were injected, there was a small R_2_* relaxivity rate decrease with poor significance in the joint space when compared to non-injected stifles (R_2_* = 64 ± 6 s^−1^ w/SPIOs; 70 ± 7 s^−1^ w/o SPIOs, p-value = 0.10) (Figs. 3 and 4).

### Labelled and unlabelled cells visualized with 3D cones MRI

The study was designed to investigate whether SPIO-labelled MSCs could be detected in osteochondral defects using 3D cones based MRI in both an acutely inflamed joint (cells injected 1 week post surgery) and in a healing joint in which the inflammation has begun to wane (cells injected 4 weeks post surgery)^30^. The acutely inflamed joint has a higher level of inflammatory cytokines and enhanced cellular infiltration, which could lead to altered host/MSC interactions. Therefore, the results of the novel MR sequences were considered at two time points. No significant differences were observed in the presence of SPIOs between the 1 week and 4 week post surgery animals with any measurement.

3D cones images are shown in Fig. 4 comparing sheep with unlabelled MSCs and SPIO-labelled MSCs in order to identify the effect of MSCs with and without SPIOs. Images at three echo times (TE = 0.03, 4.0, 16.1 ms), as well as a difference image between the first and second echo time, are shown in order to observe the possible SPIO and/or MSC induced changes more closely. No significant differences were observed at the site of the defect in joints that were injected with MSCs +/−SPIOs, nor in control joints in which no MSC were injected (Fig. 4).

### Histology and MRI

**Figure 5:**
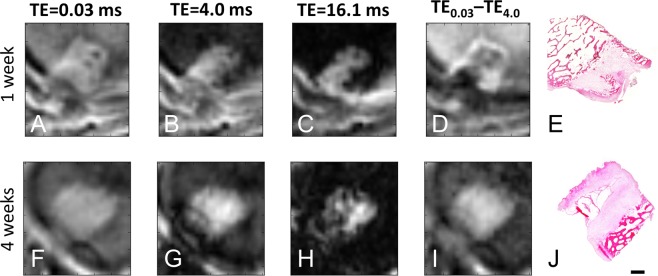
3D cones images of the osteochondral defect created in ovine distal femoral condyles (**A**–**D**,**F**–**I**) and (H&E) stained histological sections of the corresponding osteochondral defect (**E**,**J**). Each row is a different animal. Images (**A**–**E**) were obtained from an animal imaged two weeks post-surgery (one week post surgery; one week post MSC injection); images (**F**–**J**) were obtained five weeks post-surgery (four weeks post surgery; one week post MSC injection). (**A**,**F**) were obtained with TE = 0.03 ms; (**B**,**G**) were obtained with TE = 4 ms; (**C**,**H**) were obtained with TE = 16.1 ms. (**D**,**I**) are obtained by subtraction of the TE = 0.03 ms and TE = 4 ms signal images, which highlights regions that have slow signal decay [hypointense] compared to fast signal decay [hyperintense]. Excellent correlation exists between the 3D cones sequences and the histological sections, particularly in the subtracted images (**D**,**I**). No significant presence of SPIOS are detected in the defect, which would be indicated by higher signal loss in TE = 16.1 ms images. Scale bar = 500 μm.

**Figure 6:**
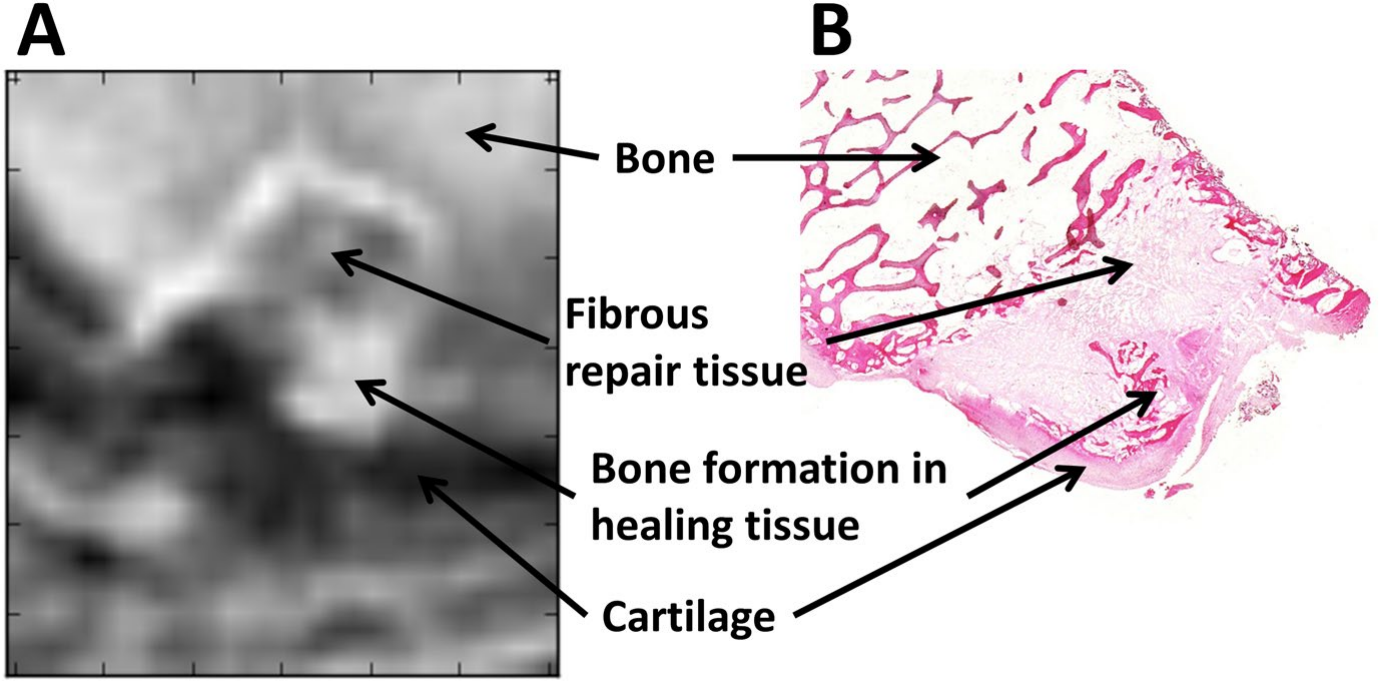
(**A**) 3D cones subtraction image of the osteochondral defect created in an ovine distal femoral and (**B**) H&E stained histological section of the corresponding osteochondral defect. These images were obtained two weeks post surgery, after SPIOs had been present for one week. The image in (**A**) represents the image subtraction between TE = 0.03 ms and TE = 4 ms images (see Fig. 6). This figure demonstrates the high degree of correlation between the MR image and the histological appearance of the tissue.

The 3D cones images matched the histology detail of the recovered osteochondral defects with good detail (Figs. 5 and 6). Within the osteochondral defect, 3D cones images at both early (0.03 ms) and late (0.16 ms) echo times had similar intensities to muscle tissue, indicating a lack of SPIOs in the defects (Fig. 5). The 3D cone images demonstrated the presence of anatomical features of the healing defects that were observed in the histological images, with the highest anatomical detail being demonstrated following post-acquisition processing (Fig. 5). A difference between the first and last UTE MR images showed features that matched well with histology, demonstrating bone, fibrous repair tissue, bone tissue formation, and cartilage (Figs. 5 and 6). Although the images between 1 week and 4 week animals appear different in Fig. 5, these features were not unique to either time point when viewed across the population. In addition, the UTE MR images show additional details with the intermediate echo times, which were not readily apparent on histological samples (Fig. 6).

## Discussion

The aim of this study was to demonstrate the utility of a novel MRI method for imaging SPIO-labelled MSCs and their subsequent effects in an ovine osteoarthritic model represented by joint defects. Signal decay increases following the introduction of SPIOs and is difficult to measure in bone, which itself has rapid signal decay. This signal loss interferes with the interpretation of MRI cell tracking with standard acquisition schemes, such as Cartesian readouts. The 3D cones images were able to reduce SPIO artefact and match histology within bone. The images also showed bone/cartilage features on intermediate echo images that were not visible on histology. The 3D cones MRI method provides excellent anatomical detail of joint defects, and reduces the signal loss from SPIO aggregation that was observed in surrounding tissues. UTE MRI methods, such as 3D cones, should be considered in future SPIO-labelled cell tracking experiments to improve visualisation and relaxation measurements.

MRI gave a clear indication of where the MSCs were located and was unable to detect MSCs in regions beyond the fat pad or synovial fluid allowing us to confirm that MSCs were not in other regions. This is clinically useful as it is not feasible to perform histological measurements that involve tissue sectioning and disruptive surgery. We conclude that if the MSCs do hone to the osteochondral defect, they may do so at a much earlier time point than a week after surgical creation of the defect. In a natural in vivio situation while differentiation and honing to the defect is possible after seven days, this would be tracking of descendent cells, due limited lifespan of MSCs.

SPIO-labelled MSCs have been previously demonstrated to be present for up to 60 days in the brain^31^, 42 days in the myocardium^32^ and 28 days in joints^33^. These^31–33^ were for longer durations that we were investigating in this study, although our study did not show SPIOs in the defects at one week after injection. A longitudinal study is suggested with earlier time points to confirm whether there are earlier honing effects. An *in vivo* longitudinal study was not performed due to insufficient access to a 3 T MRI research system that permissed live animal imaging; an ex vivo longitudinal study was not performed as that requires an initial proof of concept study to demonstrate a relationship between 3D cones MR imaging and histology. This study indicates that both earlier and longer term effects of injected MSC should be further investigated.

The signal in the lateral fat pad, synovial region between the menisci, or defect is not higher in the sheep knees injected with SPIOs compared to those without SPIOs. While SPIOs were clearly visible in concentrated areas (Fig. 2), the relaxation rates and decay ratios were lower in the three mentioned ROIs, with 3D Cones relaxation rates lower by 6–10%, although this has low significance (p-value = ~0.1). An increase of relaxation rates was seen in these areas with FSE R2 with much worse significance (p-value > 0.4). The presence of SPIOs would increase these relaxation rates above the rates in the joint without SPIOs, suggesting that there are not sufficient SPIOs remaining in these areas a week after injection.

The results of our study of 3D cones at multiple echo times demonstrates a high level of anatomical detail is retained even in the presence of SPIOs. Tracking SPIO labelled MSCs with UTE MRI has been previously reported in a rodent tumor model^27^, but has not been shown in musculoskeletal applications, nor shown in a large animal model. Standard acquisition schemes using Cartesian readouts cause bony and SPIO induced signal loss, thereby interfering with the interpretation of MRI cell MR imaging and tracking. Imaging of SPIO-labelled MSC has been reported by several authors in a number of different tissues^25–27,34^. Previous MSC SPIO imaging studies in the joints of experimental animals have typically measured T_2_* with Cartesian gradient echo where the anatomical fidelity is compromised in the presence of SPIOs^25,26^. However, imaging of SPIO-labelled cells in joints poses a imaging challenge due to the presence of dense bone, whether subchondral and osteoarthritic, and, if present, of the highly magnetic SPIOs that appears present in nearby fluid capsules. This difficulty has led to previous studies raising questions over the specificity of the sequences used for SPIO imaging in the joint^34^.

Using ovine stifles presented a number of technical challenges including the smaller size of the joint and thickness of cartilage. The dimensions of the ovine stifle region (wider in the dorsal-ventral direction and thinner in the left-right direction, when compared to human knees) required a 12-channel head coil to optimise for signal-to-noise by minimizing the coil distance to tissue. Other available coils, such as a human knee coil, were not sufficiently large for the stifle. An optimized coil, which would be similar to an abdominal coil with smaller loop dimensions, could further improve the signal-to-noise. Due to the 3D acquisition of 3D cones, additional consideration must be given for the larger required field-of-view to prevent image aliasing.

One limiting factor for the 3D cones readout is the MRI system performance, due to the intensive memory and computational requirements during both acquisition and reconstruction. Also, in order to be considered viable for future longitudinal studies, the scan time was constrained to be less than 10 minutes. Longer scanning times would result in higher resolutions, although at the time of acquisition, even higher resolutions were limited to the memory available on the MRI system, due to the computational requirements of both acquiring and reconstructing the data. The slice thickness and resolution was lower for the 3D cones readout (0.56 × 0.56 × 3.0 mm^3^) compared to the Cartesian readout (0.35 × 0.35 × 1.5 mm^3^), and the total acquisition time was nearly twice as long. At the time of experiment, the MR system has limited memory requirements for the loading of large, arbitrary gradient waveforms. These requirements limited the number of spirals used during the readout, and the achievable resolutions during acquisition. Memory is also a requirement for online reconstruction, although is less performance critical to driving the MRI system. Online reconstruction finished within five minutes of data acquisition on a DV24 GE platform. Despite these performance limitations, the 3D cones images corresponded well with histological samples.

The mechanism by which MSCs exert an influence in healthy and diseased tissues still remains unknown. Early reports described MSCs ‘honing’ to a defect and contributing to ‘building blocks’ for tissue regeneration^35^. However, *in vivo* studies using MRI tracking of SPIO-labelled MSCs have failed to provide any evidence of this honing – in both osteoarthritic models and joint defect models, with SPIO signals largely being confined to the synovium^25^, as reported in this study. Whilst MSCs may not contribute directly to the re-building of damaged tissue, a body of evidence supports a role in modifying and mediating disease. Increasing evidence strongly suggests that MSC exert a paracrine effect on resident cells^36^, possibly via the intermediary of MSC derived extracellular vesicles (EVs)^37^. EVs from MSCs have been shown to contain a number of biologically active factors and have, alone, been shown to have beneficial effects on pathology^38^. If EVs are the ‘active agent’ of MSC, then tracking and understanding their distribution within target tissues such as joints becomes, as with MSC tracking, a significant issue. The development and validation of sensitive MRI methods such as described in this study will be key to understanding MSC biology.

This study has shown that using 3D cones for tracking SPIO-labelled MSC generates MR images of high anatomical detail, evidenced by a similar image features between the MRIs and the histological evaluation of ovine joints. The superior structure detection of this method over standard Cartesian MRI acquisitions is a unique strength of UTE MRI provided by 3D cones, which we would like to use for future longitudinal monitoring of both clinical and experimental joint surface defect healing. UTE methods will enable us to further validate biological healing of small osteochondral defects, which are difficult to image due to their small size and the fast MRI signal decay rates of bones and SPIOs. Confirmation of the correlation between MRI and histology will help future studies monitor not only MSC locations, but also monitor the effects of mesenchymal stem/stromal cell healing of damaged tissues.

## Methods

### Animals

Seven mature female Welsh Mountain Sheep were used in this study with approval from from both the Animal Welfare and Ethical Review Body, University of Cambridge and the UK Home Office Project Licence number 70–7740. All methods were performed in accordance with the relevant guidelines and regulations^30^.

### Bone marrow harvest

Autologous MSCs were isolated by bone marrow aspiration from the iliac crest of anesthetized animals and collected in αMEM containing 10% FBS, 1% L-glutamine (LG), 1% antibiotic and anti-mycotic (AA) and a heparin sodium solution to prevent clotting (5000 IU/ml, Wockhardt, Wrexham, UK). The aspirate was then transported on ice for downstream MSC isolation^30^.

### Cell isolation and expansion

From the aspirate, autologous ovine MSCs were isolated by red blood cell (RBC) lysis treatment and subsequent growth occurred within αMEM media containing 20% FBS, 1% L-Glutamine and 1% AA for 1 day before further media changes. MSCs were subsequently cultured under standard cell culturing conditions in αMEM expansion media (EM; 10% FBS, 1% L-glutamine and 1% AA) until passage 2 as previously described for sheep MSC^30^.

### SPIO labelling of MSCs

MSCs were labelled with Nanomag-D *(*Micromod, Germany*)*, a commercially available 250 nm SPIO with COOH functionality and using the cell penetrating peptide P218R (gifted from the University of Nottingham), that we have previously demonstrated does not interfere with cell growth or differentiation capacity [30] and that is retained within the cells over the experimental time period [30]. Cells were labelled at a ratio of 25 µg of Nanomag (1 mg/ml) per 2 × 10^5^ cells and complexed with 1 µl (1 mM) P218R per 50 µg Nanomag. In brief, MSCs (P2) were seeded in T175 flasks at 80% confluency in EM and allowed to attach overnight. Media was then replaced with the labelling solution (consisting of serum free media (SFM) and the appropriate amount of Nanomag+P218R) and cells incubated overnight at 37 °C and 5% CO_2_ to enable efficient internalization of Nanomag. Following this, cells were washed thoroughly in PBS (3×) to remove non-internalised Nanomag. Nanomag uptake was confirmed with Prussian Blue staining of dried and fixed cells^30^.

### Surgical procedure

The left stifle joints of each animal were opened via a parapatellar approach with the animals under general anaesthesia. An 8 mm diameter, 8 mm deep, osteochondral defect was created in the medial femoral condyle (MFC) in the right stifle joints of each animal under strict asepsis. The defects were centralised in the medial femoral condyle, aligned with the medial crest of the trochlear groove and 10 mm distal to the condyle groove junction. After surgery, the joints were closed in routine fashion, and the animals were allowed to fully bear weight post-operatively^30^.

### Cell delivery

Prior to delivery, Nanomag+P218R labelled cells were stained with CM-DiI (Molecular Probes, Paisley UK) a fluorescent cell tracker as described previously^30^. 10^7^ labelled cells were subsequently re-suspended in 2 ml SFM containing 1% LG and1% AA^30^. The animals received labelled MSC injections in the left joint and unlabelled MSC injections in the right joint. Injections into the femoro-patella joint occurred at one week post-surgery (n = 4) and four weeks post-surgery (n = 6). One animal received a focal injection of MSC into the supra-patella fat pad 1 week post-surgery. Animals were humanely killed with an overdose of anaesthetic one week after MSC injection.

### Magnetic resonance imaging

Imaging was performed on the post mortem operated stifles using a 12-channel head receive-only coil on a 3.0 T MRI system (MR750 GE Healthcare, Waukesha, WI, USA). This coil provided a large volume in order to cover the length of the stifle.

Sagittal UTE images were acquired with a 3D cones k-space acquisition using a multi-echo gradient echo (mGE) sequence. The sequence repeated five k-space readouts at each TE and within a single repetition time (TR) for R_2_* quantification. The field-of-view (FOV) was 180 mm × 180 mm using a reconstruction matrix = 320 ×320, bandwidth = ±62.50 kHz, slices = 36, slice thickness = 3.0 mm, points per interleaf = 452, number of spiral interleaves = 442, averages = 1, TR = 23 ms, TEs = 0.03, 4.0, 8.1, 12.1, 16.1 ms, flip angle = 15°, and total scan time = 6 minutes 6 seconds for all echoes.

Sagittal Cartesian 3D mGE images were also obtained for quantitative R_2_* MRI, using the Susceptibility Weighted Angiography (SWAN) pulse sequence^39,40^. The scan parameters were FOV = 180 mm × 162 mm, reconstruction matrix = 512 × 460, bandwidth = ±195 kHz, flip angle = 20°, slices = 72, slice thickness = 1.5 mm, TR = 40.7 ms, using TEs = 7.0, 12.8, 18.5, 24.3, 30.1, 35.8 ms, which were the used 70% asymmetric sampling, coil acceleration (ASSET) = 2, and total scan time = 3 minutes 7 seconds.

Sagittal Cartesian 3D fast spin echo (FSE) imaging was performed with multiple MLEV (Malcolm Levitt’s composite-pulse decoupling) preparations for R_2_ mapping. The FSE preparation for R_2_ measurements consisted of a non-selective MLEV train of equally separated (+)90°–180°-(−)90° pulses^41^. The sequence parameters were FOV = 180 mm × 180 mm, reconstruction matrix = 320×320, slices = 72, slice thickness = 3.0 mm, bandwidth = ±78.08 kHz, flip angle = 20°, TR = 1920ms, TEs = 2.8, 31.7, 63.8, and 112 ms, echo train length = 45, coil acceleration (ASSET) = 2, 50% partial Fourier phase encoding (half-NEX), total scan time = 6 minutes 56 seconds.

### Image processing

Relaxation maps were calculated using a linear least-squares regression created using the Python NumPy package (NumPy Version 1.11.0, Python Software Foundation, https://www.python.org/).

The ratio of the UTE signals was also calculated as a secondary measurement of R_2_* signal decay. Cartilage has multiple R_2_ (or R_2_*) components^42^, which would not be captured by an R_2_* map using all TEs^42,43^. More intermediate TEs should be measured In order to properly measure multi-exponential decay rates with values intermediate to the first and second TE, or shorter than the first TE. The ratio of UTE signal loss is defined here such that it correlates with R_2_^*^, where the signal decay ratio presented below, R, increases with increased relaxation rates. A two-point R_2_* estimate can be obtained through the negative logarithm of these values minus one. These UTE signal ratios were calculated as

$$\begin{array}{c}{R}_{15}=\frac{\Delta {S}_{15}}{{S}_{1}}=\frac{S(T{E}_{1})-S(T{E}_{5})}{S(T{E}_{1})},\\ {R}_{12}=\frac{\Delta {S}_{12}}{{S}_{1}}=\frac{S(T{E}_{1})-S(T{E}_{2})}{S(T{E}_{1})},\\ {R}_{25}=\frac{\Delta {S}_{25}}{{S}_{2}}=\frac{S(T{E}_{2})-S(T{E}_{5})}{S(T{E}_{2})}.\end{array}$$

The means and standard deviation of each relaxation map was calculated in five regions-of-interest (ROIs), which included: the synovial joint space between the menisci; the fat pad; the bone on the tibial plateau between the cartilage and physis; a large region of dorsal muscle; and the defect containing region, or a similar region of bone in sheep without the defect. This ratio represents the signal loss between two echoes, where it is equal to unity when 100% signal loss has occurred and zero when no signal has been lost between echo times.

### Significance test

A t-test was performed on each tissue to compare between the relaxation values with and without SPIOs. Significance was considered if the t-test p-value was below 0.15.

### Histology

After imaging, the osteochondral defect sites were retrieved and decalcified in formic acid/sodium citrate over 4 weeks, prior to routine paraffin processing. Sections through the central portions of the defect were made 10 μm thick and stained with Prussian Blue and Haematoxylin and Eosin. Fluorescent and direct light microscopy was used to identify the presence of SPIOs and tissue architecture.
