# Author Correction: Ultra Short Echo Time MRI of Iron-Labelled Mesenchymal Stem Cells in an Ovine Osteochondral Defect Model

**DOI:** 10.1038/s41598-020-68531-z

**Published:** 2020-07-02

**Authors:** Joshua D. Kaggie, Hareklea Markides, Martin J. Graves, James MacKay, Gavin Houston, Alicia El Haj, Fiona Gilbert, Frances Henson

**Affiliations:** 1 Department of Radiology, University of Cambridge, Box 218, Cambridge, UK; 2 Cambridge University Hospitals NHS Foundation Trust, Addenbrooke’s Hospital, Cambridge, UK; 3 Institute of Science and Technology in Medicine, Guy Hilton Research Centre, Keele University, Thornburrow Drive, Stoke-on-Trent, ST4 7QB UK; 4 Department of Chemical Engineering, Healthcare Technologies Institute, Birmingham University, Birmingham, B15 2TT UK; 5 GE Healthcare, Amersham, UK; 6 Division of Trauma and Orthopaedic Surgery, University of Cambridge, Cambridge, UK; 7 Comparative Musculoskeletal Biology Group, Department of Veterinary Medicine, University of Cambridge, Cambridge, UK

Correction to: *Scientific Reports* https://doi.org/10.1038/s41598-020-64423-4, published online 21 May 2020

The acknowledgments section in this Article is incomplete.

“We are grateful to Ferdia A Gallagher, Stephen McDonnell and Andrew McCaskie for their support of this work. We would like to acknowledge Karin Newell for her help in preparing the specimens and James Dixan for the provision of the P21-8R peptide. This work was supported by GlaxoSmithKline, the Engineering and Physical Sciences Research Council (EPSRC), Arthritis Research UK, European Union’s Horizon 2020 research and innovation programme under grant agreement no. 761214, Addenbrooke’s Charitable Trust, and the NIHR Cambridge Biomedical Research Centre.”

should read:

“We are grateful to Ferdia A Gallagher, Stephen McDonnell and Andrew McCaskie for their support of this work. We would like to acknowledge Karin Newell for her help in preparing the specimens and James Dixan for the provision of the P21-8R peptide. This work was supported by the Engineering and Physical Sciences Research Council (EPSRC), Arthritis Research UK, European Union’s Horizon 2020 research and innovation programme under grant agreement no. 761214, Addenbrooke’s Charitable Trust, and the NIHR Cambridge Biomedical Research Centre. JDK and JM received stipendiary support from GlaxoSmithKline.”

